# Immunogenic Cell Death: Can It Be Exploited in PhotoDynamic Therapy for Cancer?

**DOI:** 10.1155/2013/482160

**Published:** 2012-12-30

**Authors:** Elisa Panzarini, Valentina Inguscio, Luciana Dini

**Affiliations:** Department of Biological and Environmental Science and Technology (Di.S.Te.B.A.), University of Salento, Via per Monteroni, 73100 Lecce, Italy

## Abstract

Immunogenic Cell Death (ICD) could represent the keystone in cancer management since tumor cell death induction is crucial as well as the control of cancer cells revival after neoplastic treatment. In this context, the immune system plays a fundamental role. The concept of Damage-Associated Molecular Patterns (DAMPs) has been proposed to explain the immunogenic potential of stressed or dying/dead cells. ICD relies on DAMPs released by or exposed on dying cells. Once released, DAMPs are sensed by immune cells, in particular Dendritic Cells (DCs), acting as activators of Antigen-Presenting Cells (APCs), that in turn stimulate both innate and adaptive immunity. On the other hand, by exposing DAMPs, dying cancer cells change their surface composition, recently indicated as vital for the stimulation of the host immune system and the control of residual ill cells. It is well established that PhotoDynamic Therapy (PDT) for cancer treatment ignites the immune system to elicit a specific antitumor immunity, probably linked to its ability in inducing exposure/release of certain DAMPs, as recently suggested. In the present paper, we discuss the DAMPs associated with PDT and their role in the crossroad between cancer cell death and immunogenicity in PDT.

## 1. Introduction

The plain success of cancer therapies crucially depends on the synergic interaction between immune cells and dying/dead cancer cells. The ideal cancer treatment should merge the direct cytotoxic action on tumor cells with potent immunostimulatory effects based on the recognition of molecular immunogenic determinants on dying cells by immune cells. Indeed, anticancer immune responses may contribute to the control of the neoplastic disease after cancer modalities since they help to eliminate residual cancer cells or maintain micrometastases in a stage of dormancy. The capability of a cancer treatment to elicit Immunogenic Cell Death is clinically relevant since it is associated with an anticancer immune response that reinforces the therapeutic effect of the therapy. The immunogenicity of the dying cancer cells involves subtle changes in their surface proteome and the secretion of soluble molecules known as Damage-Associated Molecular Patterns (DAMPs) allowing their immunogenic recognition by immune effectors. 

In recent times, more and more efforts are addressed to associate particular DAMPs with a specific cell death pathway or with particular stress agents able to induce Immunogenic Cell Death (ICD) in cancer cells. One such therapeutic modality certainly associated with DAMPs is PhotoDynamic Therapy (PDT). In the present paper, we collect data regarding DAMPs related to PDT, primarily focusing on the ability of these molecules to function as ICD effectors in PDT. 

## 2. Emerging Hallmarks of Cancer 

During their evolution to the malignant state, tumor cells progressively evolve multiple ploys to carry out their intrinsic fateful program. Particularly, cancer cells acquire six distinctive and complementary biological capabilities allowing tumor growth and metastatic dissemination. These include self-sufficiency in growth signals, insensitivity to growth suppressors, circumventing cell death mechanisms, limitless replicative potential, sustained angiogenesis, and tissue invasion and metastasis [1]. Cancer cells do not need stimulation from external growth factors to grow and divide since they can generate their own growth signals sustaining chronic proliferation. Unlike normal cells whose growth is kept under control by inhibitors in the surrounding environment, in the extracellular matrix and on the surface of neighboring cells, tumor cells are generally resistant to growth-preventing signals becoming masters of their own destinies. They are able to bypass apoptosis, the preferential form of Programmed Cell Death (PCD) induced by conventional cancer therapies, by the loss of Tumor Protein 53 (TP53) tumor suppressor function, the upregulation of antiapoptotic regulators (Bcl-2, Bcl-xL) or of survival signals (Igf1/2), the downregulation of pro-apoptotic factors (Bax, Bim, Puma), or the short-circuiting of the extrinsic ligand-induced death pathway. Normal cells undergo a limited number of successive cell growth-and-division cycles, since their proliferation is subjected to two distinct barriers: senescence, a viable state characterized by an irreversible arrest in proliferation limiting the lifespan of mammalian cells, and crisis, which involves cell death. On the other hand, cancer cells escape these barriers and they are capable of indefinite growth and division (immortality). In fact, the immortal cells present damaged telomeres, the regions of repetitive nucleotide sequences at each end of a chromosome, that are centrally involved in this unlimited proliferation capability [2]. In order to progress, cancer cells must turn on a blood supply, generated by the process of angiogenesis, ensuring a continual provision of oxygen and other nutrients. Angiogenesis is balanced by inducers, such as vascular endothelial growth factor (VEGF) and acidic and basic fibroblast growth factor (FGF 1/2), and inhibitors, including thrombospondin-1. Thrombospondin-1 is regulated by p53, therefore the loss of p53 can allow angiogenesis. Tumor cells can migrate from their origin site to invade surrounding tissue and metastasize to distant body areas through a multistep process, referred to as invasion-metastasis cascade [3], characterized by a succession of cell-biologic changes. These include (1) local invasion, then (2) intravasation by cancer cells into nearby blood and lymphatic vessels, (3) transit of cancer cells through the lymphatic and hematogenous systems, followed by (4) escape of cancer cells from the *lumina* of such vessels into the parenchyma of distant tissues (extravasation), (5) the formation of small nodules of cancer cells (micrometastases), and finally the growth of micrometastatic lesions into (6) macroscopic tumors (colonization).

The acquisition of the six functional capabilities allowing cancer cells to survive, proliferate, and disseminate is made possible by two enabling characteristics: genome instability, which generates random mutations, such as chromosomal rearrangements, driving tumor progression; and inflammation by innate immune cells, which results in tumor-promoting consequences. Indeed, the immune system both antagonizes and enhances tumor development and progression, playing dichotomous roles. In the last decade two emerging hallmarks have been added to this list: reprogramming of energy metabolism in order to most effectively support neoplastic proliferation and evading immune destruction by T and B lymphocytes, macrophages, and natural killer (NK) cells. Particularly, the abilities to replicate in a chronically inflamed microenvironment, to evade immune recognition, and to suppress immune reactivity enable neoplastic cells to escape the immune responses [1].

The poor antitumor immunity and the escape to the innate and adaptive immune responses are based on the downregulation of tumor cell Major Histocompatibility Complex (MHC) I and costimulatory molecules, alteration of DCs and macrophages function in tumor tissue, regulatory T cells induction, and tumor-mediated immune cell death [4]. Besides, tumor resistance may also be a consequence of the altered expression of oncogene-coded proteins, as demonstrated in ovarian carcinoma-derived cells expressing low levels of HLA class I surface antigens and decreased or absent HLA-A2 expression [5]. Also dysregulation of various components of the MHC class I Antigen Processing Machinery (APM) may avoid the recognition of tumor cells by CD8+ T cells [6]. 

The long-standing concept of immunosurveillance implying the constant monitoring of cells and tissues by an ever-alert immune system able to recognize and remove nascent transformed cells [7] has been abandoned in favor of the cancer immunosurveillance acting as a component of the cancer immunoediting. Particularly, cancer immunoediting, which represents a refinement of the original cancer immunosurveillance hypothesis, plays a dual role in promoting host protection against cancer and facilitating tumor escape from immune destruction. It is responsible for both eliminating tumors and sculpting the immunogenic phenotypes of tumors as they develop. This process consists of three phases that are collectively denoted “the three Es of cancer immunoediting”: elimination, equilibrium, and escape. Elimination corresponds to immunosurveillance; equilibrium represents the process by which the immune system iteratively selects and/or promotes the generation of tumor cell variants with increasing capacities to survive immune attack; escape is the process wherein the immunologically sculpted tumor expands in an uncontrolled manner in the immunocompetent host [8].

In the clinical management of the neoplastic disease, the ideal therapeutic strategy should combine the restoration of cancer cell death and the enhancement of the immunological recognition of tumor cells [9]. This may be achieved by avoiding cancer modalities mediating immunosuppressive side effects and favoring therapies able to induce ICD, which represents a novel possibility to attack neoplasia with the specificity of the immune system [10]. 

It is still unclear under which circumstances cellular demise induces an immune response against dying tumor cells or rather it remains immunologically silent. The classical notion that apoptotic cell death is poorly immunogenic (or even tolerogenic), whereas necrotic cell death is truly immunogenic has been recently invalidated since it does not withstand experimental verification, at least in models of tumor vaccination [11, 12]. Indeed, tumor vaccination studies in mice demonstrate that some apoptosis-inducing regimens induce immune-dependent tumor regression whereas others do not, suggesting an unsuspected heterogeneity in the biochemical pathways leading to apoptotic cell death [13].

The immunogenicity of dying cells is mediated by changes in the composition of the cell surface and the secretion of soluble molecules allowing the immune effectors, primarily dendritic cells (DCs), to sense immunogenicity [14]. Intracellular molecules, categorized as Damage-Associated Molecular Patterns (DAMPs), also known as alarmins, normally hidden within live cells, are released from or exposed at the surface of dying cell determining DCs activation and maturation, antigen processing, and T cell activation (see below). 

The appealing idea of immunogenic cancer cell death demands screening of newer anticancer agents/modalities capable of sustaining a particular spectrum of DAMPs. Indeed, a chemotherapeutic agent-specific cancer ICD modality presents the potential to induce *in vivo* an “anticancer vaccine effect” by merging tumor cell kill and antitumor immunity within a single paradigm.

## 3. PhotoDynamic Therapy: Basic Principles and Applications

One recent therapeutic modality endowed with a known association with certain DAMPs is PhotoDynamic Therapy (PDT), a Food and Drug Administration (FDA)-approved clinical protocol for the treatment of several malignant and nonmalignant diseases [15]. PDT presents multiple advantages over “classical” anticancer regimens, such as surgery, ionizing radiation, and chemotherapy: it is minimally invasive, it has low mutagenic potential, low systemic toxicity and it specifically targets tumor areas over normal tissue [16, 17].

It is a two-step procedure involving the administration of a tumor-localizing photosensitizer (PS) and its subsequent activation by light of specific wavelength. PDT utilizes the destructive power of Reactive Oxygen Species (ROS) generated *via* photophysical/photochemical reactions by the interaction between visible light, PS, and tissue molecular oxygen, the three main components of the photodynamic reaction, to elicit cancerous cells obliteration [18]. 

Efficient photosensitization primarily depends on the PS physico-chemical properties, including chemical purity, selectivity for cancer cells, chemical and physical stability, short time interval between the drug administration and its accumulation within tumor cells, activation at wavelength with optimal tissue penetration, and rapid clearance from normal tissues [19], and it is related to the amount of oxygen within the tumor area that, in turn, depends on the tissue oxygen concentration [20]. Photodynamic treatment also strictly relies on the light source and light delivery, whose choice is affected by tumor location, light dose delivered, and PS used. Lasers, lamps, and Light Emitting Diodes (LED) are all light sources employed in PDT. Conversely to lamps, lasers are typically near-monochromatic enabling the exact selection of wavelengths and the precise application of light. On the other hand, the main characteristics of LED use are price and versatility in light delivery on difficult anatomic area [21].

The photodynamic reaction is based on photophysical and photochemical processes [22]. Upon visible light irradiation, the PS in its ground state is activated to the short-lived single excited state and it can lose its energy by emitting fluorescence or vibrational energy (photophysicalreaction). The excited singlet state PS may also undergo a process known as intersystem crossing to form a relatively long-lived excited triplet state (photochemical reaction), which may interact with surrounding molecules resulting in two types of photooxidative reactions exploited in PDT. In type I photochemical reaction, the PS excited triplet state directly reacts with a substrate, such as the cell membrane, and it transfers an electron or hydrogen atom producing radical forms. These intermediates may further react with oxygen to form peroxides, superoxides ions, and hydroxyl radicals (known as ROS), initiating free radical chain reactions. Alternatively, type II photochemical reaction involves the direct transfer of triplet PS energy to molecular oxygen to form excited-state singlet oxygen (^1^O_2_), the most important reactive species in PDT-mediated cytotoxicity [23]. The two types of photochemical reactions can simultaneously occur and their ratio depends on the type of PS, substrate, and oxygen concentration.

PDT-mediated tumor destruction is multifactorial: (1) direct tumor cells killing, (2) vasculature damage, and (3) rapid recruitment and activation of immune cells favoring the development of antitumor adaptive immunity [18, 24, 25].

Particularly, cancer cells can respond to photodynamic injury by initiating a rescue response and/or succumbing to multiple cell deaths. Three distinct mechanisms have been recognized to contribute to PDT-mediated tumor destruction: apoptosis, necrosis, and autophagy [26]. Apoptosis is the preferential PCD induced by the exposure of many photosensitized cell types to toxic agents, such as ROS. Apoptosis can be activated both in a caspases-dependent and independent manner. Particularly, PDT leads to activation of the several apoptotic pathways: extrinsic or death receptor pathway, implying the binding of death ligands to their specific cell surface death receptors (e.g., FasL/FasR, TNF-*α*/TNFR1, Apo3L/DR3, Apo2L/DR4, and Apo2L/DR5) ending in caspase 8 activation; intrinsic or mitochondrial pathway, involving caspase 9 activation and release of cytochrome c into the cytosol; ER stress-mediated pathway, mediating the cleavage of caspase 12; and caspase independent pathway, triggered by mitochondrial proapoptotic proteins, for example, AIF (Apoptosis Inducing Factor) and EndoG (Endonuclease G), able to induce apoptosis without caspase involvement by translocating to the nucleus where they generate DNA fragmentation (reviewed in [18, 26]). Master regulators of apoptotic machinery are Bcl-2 family proteins, comprising both anti- and proapoptotic members [27]. PDT is able to induce photoxidation of Bcl-2 antiapoptotic proteins and activate the proapoptotic members of the family [28].

If apoptosis is the preferential cell death mechanism induced by PDT, a switch from apoptosis to necrosis strictly depends on PDT dose in term of light dose and PS concentration. Indeed, high PDT dose tends to cause cell death by necrosis, while low photodynamic regimen induces apoptotic cell death.

The role of autophagy in PDT-treated cells is controversial, since it plays a role in either inhibiting or stimulating cell death following photodynamic treatment [29, 30]. Although autophagy is generally thought of as a cell survival strategy, the high reactivity of photogenerated ROS can commit tumor cells to their final demise [31]. Generally, autophagy plays a prosurvival role in tumor cells capable of apoptosis; conversely, it promotes cancer cell death in apoptosis-deficient cells.

In some experimental PDT protocols, the specific inhibition of one of these three cell death mechanisms does not impair the activation of the others, suggesting their independent onset in PDT, which is able to ensure a long-term tumor photokilling [32, 33]. 

Tumor eradication is also mediated by strong PDT-induced inflammatory and immune reactions ending in the rapid recruitment of immune cells to neoplastic sites. Several reports suggest the infiltration of lymphocytes, leukocytes, and macrophages into the photosensitized tissue activating an immune response that consequently eliminates surviving cancer cells escaped to the direct PDT effects [34, 35]. 

The considerable beneficial immunomodulatory potential of PDT represents an exploitable plot in terms of cancer disease management. High-inflammatory PDT regimens induce acute inflammation characterized by increased expression of proinflammatory cytokines [36], adhesion molecules E-selectin and ICAM-1, and the rapid accumulation of leukocytes into the treated tumor area [37]. PDT enhancement of antitumor immunity appears to involve the stimulation of DCs by dying tumor cells [38]. Indeed, the incubation of photosensitized tumor cells with immature DCs induces an enhanced DC maturation, activation, and ability to stimulate T cell activation [39].

## 4. Immunogenic Cell Death: New Concept in**** Cancer Therapy Outcome

The intrinsic Achille's heel of conventional cancer regimens, that is, chemotherapy or radiotherapy, relies on their inability to eradicate all tumor cells. However, the knowledge of cancer immune hallmarks could be exploited to stimulate immune system and, consequently, to favour the patient, by designing therapeutic regimens able to elicit the immune reactivity and counteract immune suppression. In fact the immunogenicity of the succumbing tumor cells could drive a strong immune response against cancer cells survived to therapy [40]. Indeed, in response to anthracyclins (e.g., doxorubicin and mitoxantrone), oxaliplatin and ionizing irradiation, cancer cells die triggering a tumor-specific immune responses [41]. Altogether, these observations support the Immunogenic Cell Death (ICD) concept [42]. Particularly, the signals delivered by immunogenic dying cells function as antigens stimulating the crosstalk between DCs and T cells, that in turn mediates immunogenic *impetus* [43]. 

### 4.1. The Effectors of ICD: Alarmins

The relocation, release, and/or plasma membrane exposure of intracellular proteins by dying cancer cells are the key mechanisms in ICD. These intracellular molecules, known as alarmins, are categorized as DAMPs, and they are functionally similar to Pathogen-Associated Molecular Patterns (PAMPs), including bacterial and viral nucleic acids, fungal *β*-glucan and *α*-mannan cell wall components, the bacterial protein flagellin, components of the peptidoglycan bacterial cell wall, and lipopolysaccharide (LPS) from Gram-negative bacteria [44]. 

DAMPs are normally retained within healthy cells and are extracellularly relocated in damaged/dying cells acquiring immunostimulatory/immunomodulatory properties when they interact with both intracellular and membrane-bound Pattern-Recognition Receptors (PPRs), for example, RIG-I-like receptors (RLRs), the NOD-like receptors (NLRs), and Toll-like receptors (TLRs). The diversity of DAMPs is related to the type of cell death, cell type, and tissue injury [44]. 

A multitude of immunogenic factors has been identified: Endoplasmic Reticulum (ER) protein calreticulin (CRT) [45], several members of the Heat Shock Proteins (HSPs) family [46–48], High-Mobility Group Box 1 (HMGB1) [49], end-stage degradation products (e.g., ATP, DNA and RNA, uric acid) [50–52], S100 proteins [53], and sphingosine [54]. Depending on the stage and relocation place, these molecules can be divided in three groups: (1) DAMPs exposed on plasma membrane; (2) DAMPs secreted extracellularly and (3) DAMPs produced as end-stage degradation products.

Secreted DAMPs can be in turn classified on the basis of the release mechanism: DAMPs passively released (e.g., during necrotic cell death), DAMPs released in a pulsatile manner (e.g., during apoptotic cell death), and DAMPs released by a noncanonical pathway, upon induction by activated immune cells [55]. 

Moreover, considering origin and mechanisms of action, the proinflammatory DAMPs can be classified as those that directly stimulate the immune cells and those that induce DAMPs generation exerting a bystander effect on extracellular molecules [56]. 

Furthermore, other signals, regarded as “atypical DAMPs”, are being studied as alarmins, like “whole organelle-based danger signals”, for example, complete mitochondria able to activate the NLRP3 inflammasome [57, 58], extracellular matrix compounds (e.g., hyaluronan, heparin sulphate, and degraded matrix constituents) [59], and signals/structures not yet fully characterized, for example, tumor cell-derived exosomes [60, 61]. 

In the following paragraphs, we summarize the characteristics and the translocation/release mechanisms of the main DAMPs acting as effectors of ICD.

**Calreticulin**

Calreticulin (CRT) is a 46 kDa Ca^2+^-binding protein prevalently located in ER lumen, where it acts in proper folding of proteins, by interacting with ER-resident disulfide isomerase ERp57 and calnexin (CNX) [62], and in Ca^2+^ homeostasis/signaling regulation [63]. Moreover, in ER, the CRT participates in MHC class I molecule assembly and loading of the antigen peptides onto the MHC class I molecule [64].

The CRT also resides in the nuclear envelope lumen, where it regulates nuclear protein transport [65] and signaling *via* nuclear steroid receptors and integrins [66], and in the cytoplasm. Probably, cytoplasmic CRT regulates cell adhesion, translation, gene expression, and nuclear export [67].

It has been demonstrated that CRT is also involved in cardiac development and adipocyte cells differentiation [68].

The dynamic exposure of CRT (ecto-CRT) on plasma membrane marks the cell for ICD [45]. Few studies have been performed in order to understand the mechanism regulating CRT translocation, that seems to unfold through three modules, that is, ER stress induction, apoptosis trigger, CRT translocation. Ecto-CRT translocation depends on the stress inducer and it certainly involves ROS-based ER stress; however, the ER can be or not the main inducer target, as it has been recently speculated by Garg et al. [69]. The well-known inducers of Immunogenic Cell Death, that is, anthracyclines, mitoxantrone, and oxaliplatin [45], primarily localize in the nucleus, inducing apoptosis upon DNA replication and repair damage, and only a fraction of them targets ER, producing ROS-based ER stress, indispensable to elicit immunogenicity during cell death. Similarly, inducers that preferentially localize ER, such as Hypericin, a molecule used in a particular cancer protocol, that is, PDT, are able to induce immunogenic apoptosis upon photo-oxidative ER (phox-ER) stress [70].

In anthracycline-based ecto-CRT translocation, ER stress induction ignites the ER stress response through Phosphorylation of eukaryotic Initiation transcription Factor 2*α* (eIF-2*α*-P) *via *serine/threonine kinase PERK (Protein kinase RNA-like Endoplasmic Reticulum Kinase) activation. The consequent apoptosis induction involves caspase 8, Bax and Bak Bcl-2 family members and the ER protein BAP31. CRT translocation on plasma membrane occurs in a SNARE-dependent exocytosis, based on a “CRT/Erp57 cotranslocation module” [71].

Conversely, the pathway orchestrating ecto-CRT translocation in phox-ER stress-induced immunogenic apoptosis only requires PERK and Bax/Bak, but it is independent on eIF-2*α*-P and caspase-8 [70].

Moreover, the cotranslocation of CRT with ERp57, that has been described in immunogenic apoptosis, is probably not a universal phenomenon strictly necessary for the immunogenic outcome in cancer therapies. In fact, Garg et al. [70] describe the first ERp57-independent CRT exposure upon phox-ER stress.

Plasma membrane exposed CRT facilitates the engulfment of tumor dead cells by DCs, ensuring their immunogenicity [45]. A series of studies suggests that ecto-CRT exposure occurs in apoptotic anthracyclines, oxaliplatin, UVC, and *γ*-radiation induced cancer cells [45, 72]. These cells subcutaneously injected into syngenic immunocompetent mice boost a strong anticancer immune responses, also protecting against recurrence [45, 72, 73]. Immunogenic response to tumor antigens increases in patients with Acute Myeloid Leukemia (AML) expressing CRT on cell surface of malignant blasts [74]. Moreover, Pekarikova and coworkers [75] demonstrate the presence of high percentage of anti-calreticulin antibodies in the serum of patients with gastrointestinal cancer pathology suggesting the cell surface expression of CRT on cancer cells, able to act as a target for B-cell immunogenic response.

The role of anti-CRT antibodies in cancer is enigmatic, since the translocation of CRT onto cell surface and/or its release in extracellular environment elicit an autoimmune reaction also in cancer pathologies [76–78] playing a negative role in antitumor defence [79, 80]. Probably, anti-CRT antibodies can interact with CRT engaging peptides exposed on the tumor cells surface with consequent decrement of cell immunogenicity or they can bind CRT peptides presented by MHC on APC preventing T cell response. The ecto-CRT exposure occurs before the specific morphological, that is, phosphatidylserine (PtdS) exposure on the outer leaflet of plasma membrane, and biochemical, that is, mitochondrial transmembrane potential depolarization, apoptotic signs [71]. The identity of the surface receptor docking ecto-CRT is not well known. The ecto-CRT colocalizes with PtdS on plasma membrane cholesterol rich GM-1 ganglioside-containing rafts [81]. It has been demonstrated that, in phox-ER stress, ecto-CRT surface docking does not depend on the correct organization of lipid rafts and occurs *via* the Low-density Receptor-related Protein 1 (LRP1) or CD91 molecule [70].

The extracellular CRT interacts with professional phagocytes plasma membrane CD91 internalization receptor forming a functional complex that drives engulfment of apoptotic died cells by stimulation of Rac-1 in phagocytes [82]. Further, ecto-CRT can also interact with thrombospondin [83], C1q and mannose binding lectin (MBL) [84], ficolin-2 and ficolin-3 [85, 86], and Surfactant Proteins (SP) A and D [82].

The receptor on DCs surface mediating engulfment by binding CRT-exposed on cancer cells is still obscure. Candidate receptors include scavenger receptor A [87], scavenger receptor class F, member 2 [88], and CD91 [89].

Intriguingly, ecto-CRT has been observed on the plasma membrane of the immune system cells, that is, monocyte-derived macrophages [90], DCs [91], resting and activated T-cells [92]. For instance, ecto-CRT on DCs plasma membrane interfaces cancer cells and hosts innate immune system by interacting with tumor-associated antigens like NY-ESO-1 [91]. If the presence of CRT on surface of immune cells, that is, macrophages, DCs and T-cells, effectively mediates an “anticancer vaccine effect” is still unclear.

**Heat Shock Proteins Family Members**

Inducible Heat Shock Proteins (HSPs) are a class of chaperone proteins ensuring the correct folding and subcellular compartments transport of newly synthesized proteins and the refolding or degradation of stress-accumulated misfolded ones [93].

Under stress conditions, intracellularly located HSPs are overexpressed and they can be translocated to plasma membrane surface and/or they can be also released into the extracellular environment. At least two members of HSPs family, HSP70 and HSP90, can be expressed at the surface of plasma membrane and the change of cellular localization plays a dual role in cancer [94]. In fact, intracellular, cytoplasmic and organelles-located, overexpressed HSP70 and HSP90 exert a cytoprotective role by apoptosis inhibition [93], augmenting cancer cell survival; conversely, HSPs exposure suppresses tumor by attracting innate immune system cells.

Particularly, HSP70 inhibits (a) the apoptosome complex formation needful for postmitochondrial caspase activation by interacting with Apaf-1 [95]; (b) the caspase-independent apoptosis by blocking AIF translocation from mitochondria to the nucleus [96]; (c) the proapoptotic transcription factor p53 [97] or JNK1 or ERK stress kinases [98]; (d) the Bax-dependent release of proapoptotic factor from mitochondria by blocking mitochondrial outer membrane Bax translocation [99]. On the other hand, HSP90 can negatively affect apoptosis by (a) interacting with Apaf-1 and consequently blocking the apoptosome formation [100]; (b) interacting with Akt that, in turn, leads to inactivation of proapoptotic Bad protein and caspase-9, and to activation of NF*κ*B apoptosis inhibition mechanism [101]; (c) inhibiting the action of calpains.

When HSP70 and 90 move from intracellular side to plasma membrane upon stress conditions, for example, oxidative stress, irradiation, serum deprivation, and chemotherapeutics drugs, they elicit a potent immunostimulatory activity. Particularly, in hyperthermia induced surface HSP enriched melanoma [102] and colon carcinoma cells [103], HSP70 and 90 act as DAMPs determining the immunogenicity of dying cancerous cells. HSP70 tightly associates with PtdS on plasma membrane, accelerating apoptosis as reported in PC12 tumor cells [104]. The immunostimulatory effect of ecto-HSP70 and 90 is based on their ability to interact with several APC surface receptors [105], for example, CD91, LOX1, and CD40 [106], and to elicit CD8+ T-cell response by participating in cross-presentation of tumor-derived antigens on MHC class I molecules [107]. This process is very needful in mouse models [108]. Antigen processing and cross-presentation of HSP-linked peptide involve a complex formed by Toll-Like Receptors (TLRs) and CD14 [109, 110]. TLR4 activates NF*κ*B pathway in DCs that in turn induces the release of proinflammatory cytokines, such as TNF*α*, IL-1*β*, IL-12 and IL-6, and Granulocyte Macrophage Colony-Stimulating Factor (GM-CSF) [109–112]. Besides, HSP70 can promote DCs maturation by upregulating CD86 and CD40 [113] and NKs activation by interacting with various NKs surface localized inhibitory/activating receptors, such as CD94/NKG2A [114] and CD94, respectively, [115].

A number of human and animal tumor cells, that is, colon, pancreas, breast neck and head tumors [102, 103], and acute myeloid leukemia cells [116], express HSPs on their plasma membrane upon heat shock treatment.

The immunogenic potential of HSPs also occurs when they are secreted in the extracellular environment. In fact, high levels of HSP70 and 90 have been detected both *in vitro* and *in vivo*. Particularly, in humans, several stress conditions, such as inflammation, bacterial and viral infections, and cancer diseases, lead to HSP70 and 90 presence in the serum of cancer patients. *In vitro*, the supernatants of the cultured of APCs and tumor cell lines contain members of HSPs family whose release occurs following exogenous stress, that is, proinflammatory cytokines [117].

HSPs surface translocation mechanisms and exposed HSPs derivation, that is, ER, cytosol or both, are still unknown. Also the membrane anchorage and the release process are not completely understood. Several mechanisms have been suggested to explain these phenomena including tumor cell necrosis and apoptosis and, recently, active release from viable tumor cells.

Since cytosolic HSPs do not contain the leader peptides enabling membrane localization, it has been speculated that other proteins possessing transmembrane domain shuttle HSPs from the cytosol to the plasma membrane or that HSPs directly interact with lipid components of plasma membrane. It has been demonstrated, in PC12 cells, that HSP70 associates with PtdS, supporting the hypothesis that the transport of HSP70 from inside the cell to the outer plasma membrane leaflet involves a flip flop mechanism similar to the PtdS one [104].

An active release of HSP70 along with BAG family molecular chaperon regulator 4 (Bag-4) *via *binding on surface of exosomes, endosome-derived vesicles 30–100 nm sized playing a dual role in cancer pathogenesis [118], has been observed in viable human colon and pancreatic carcinoma cells. Hsp70/Bag-4 surface-positive exosomes elicit migratory and cytolytic activity of NKs [119]. Also 4T1 breast adenocarcinoma and K562 erythroleukemic cells stimulated with IFN-*γ* increase exosomal export of HSP70 inducing IL-12 release by DCs [120]. Similarly to tumor cell lines, human peripheral blood mononuclear cells (PBMCs) in both basal and stress-induced (heat shock at 40 or 43 degrees C for 1 h) states release HSP70-containing exosomes [121].

Taking into account all the above reported contents, HSPs are reported as DAMPs. Recently, Eden and coworkers [122] argue against the role of HSPs as DAMPs on the basis of the criteria suggested by Kono and Rock in 2008 [123]. These criteria are considered essential in order to classify a particular molecule as DAMP in terms of biological outcomes: a DAMP should be active and a highly purified molecule; its biological effect should not be owing to contamination with microbial molecules; it should be active at concentrations present in pathophysiological *status*; its selective elimination or inactivation should ideally inhibit dead cells biological activity both *in vitro* or *in vivo* assays. HSPs do not satisfy the first two criteria, since, due to their chaperone nature, HSPs engage other molecular structures and they are easily contaminated with microbial products [122].

On the other hand, TLR2 and TLR4, the most credited receptors for HSP60 and HSP70, respectively, are not strictly proinflammatory [124]. Moreover, experiments performed with DCs cultured in the presence of HSPs, both in murine [125] and human models [126, 127], demonstrated no stimulatory effects on DCs activation.

Then, it has been speculated that the HSPs might function as carriers of DAMPs rather than DAMPs, [116, 128].

**High Mobility Group Box 1 (HMGB1)**

The HMGB1 is a 29 kDa nucleus localized and nonhistone chromatin bound protein, also known as amphoterin. It affects different nuclear functions, that is, transcription and nucleoprotein complexes assembly. When it is actively secreted by inflammatory cells or passively released by necrotic cells [129], it acquires immunological potential based on its redox status [130] constituting a crucial step in the activation of APCs [49]. Three types of HMGB proteins exist: HMGB1 ubiquitously expressed [131], and HMGB2 and HMGB3 mostly expressed during embryogenesis and restrictively in adult-stage [132, 133]. Moreover, HMGB1 actively secreted by IL-1*β*, TNF or LPS-activated macrophages and monocytes is molecularly different from that passively released by the necrotic cells, since it is acetylated on several specific lysine residues [134].

Extracellular HMGB1 activates macrophages and DCs [135, 136] and burst neutrophil recruitment [137] by binding to a range of receptors, including TLR2, TLR4, and RAGE (Receptor for Advanced Glycation Endproducts) [135].

HMGB1 has several unique roles in cancer. Its expression is very important in lymphoma, melanoma and breast, cervix, colon, liver, lung, and pancreas cancer cells; further, its serum level significantly increases [138].

The HMGB1 release as DAMP is widely demonstrated in necrotic cancer cells [139]. Recently, also apoptotic [41] and autophagic [140] cancer cells might release HMGB1 at some points in their respective execution phases.

The HMGB1-DNA binding dictates the time and the occurrence of release. Since nuclear DNA is released in a time-dependent manner following apoptosis induction [141] and since during apoptosis HMGB1-DNA binding increases, apoptotic cells can release DNA as well as HMGB1 during later stages, for example, secondary necrosis [130, 142, 143]. In contrast to inflammatory response initiated by necrotic cells [139], ROS produced and HMGB1 released by apoptotic cells promote tolerance [130].

Recently Liu and coworkers [144] found that HMGB1 release after vincristine (VCR), cytosine arabinoside, arsenic trioxide adriamycin (ADM) chemotherapy treatment is a critical regulator of autophagy in HL-60 and Jurkat leukemia cells and a potential drug target for therapeutic intervention. In fact, HMGB1 contributes to chemotherapy resistance through autophagy regulation [145]. It has been demonstrated that leukemia cells sensitivity to chemotherapeutic agents increases by inhibiting HMGB1 release; conversely, leukemia cells resist to cell death by overexpressing HMGB1; finally, pretreatment with exogenous HMGB1 increases leukemia cells drug resistance [145]. Moreover, HMGB1-mediated autophagy depends on Beclin-1 regulation carried out by HMGB1 itself and it requires PI3KC3-MEK-ERK pathway. In fact, Liu et al. [144] found that HMGB1 increases Beclin 1/class III phosphatidylinositol 3-kinase (PI3KC3) and suppresses Beclin 1/Bcl-2 interaction, promoting vesicle nucleation. HMGB1 also contributes to phagophore membrane elongation and autophagosome formation by promoting Atg12-Atg5-Atg16 complex formation and by enhancing the accumulation of the Atg12-Atg5-Atg16 complex with LC3.

Finally, the redox state controls HMGB1's function in promotion of autophagy. In particular, reducible HMGB1 decreases cell injury/death in cancer cells by interfering with Beclin 1, whereas oxidized HMGB1 enhances cell injury/death in response to anticancer agents [146].

**End-Stage Degradation Products**

Upon loss of membrane integrity during primary or secondary necrosis, intracellular substances normally retained within cells are released. These end-stage degradation products, including uric acid, RNA, genomic double-stranded DNA, nucleotides (ATP) and nucleosides (adenosine), exert immunostimulatory effects on macrophages and DCs [50, 147, 148]. The uric acid is the end product of purine metabolism in uricotelic mammals, identified as a major alarmin released by injured cells since it elicits inflammation by enhancing CD8+ [149] and CD4+ [150] T cell responses towards particulate antigens. Plasma membrane collapse leads to release of (1) RNA molecules, interacting with TLR3 on DCs [51]; (2) double-stranded DNA, stimulating both macrophages and DCs [50]; (3) nucleotides, triggering maturation of DCs [147] through activation of the NF*κ*B signaling [151]. Parallel to passive release, ATP can be liberated into the extracellular space by voltage-gated hemichannels such as pannexin 1 or connexin [152] and/or vesicular exocytosis [153].

**Inflammatory Cytokines**

Necrotic cells can also elicit an inflammatory response by active or passive secretion of inflammatory cytokines, that is, IL-1*α* e IL-6. In particular, Eigenbrod et al. [154] demonstrated that IL-1*α* is passively released by necrotic cells and it induces, in peritoneum mesothelial cells, secretion of CXCL1, leading to neutrophilic inflammation. Conversely, necrotic cells actively release proinflammatory cytokine IL-6 by upregulating NF*κ*B and p38MAPK [155]. End-stage degradation products modulate their recognition by the immune system cells *via* the pentraxins, a family of innate immunity receptors [156].

### 4.2. ICD: Immunogenic Signals Delivery Spatiotemporal Pattern

ICD can be triggered by a panoply of anticancer *stimuli*, including Photodynamic Therapy, anthracycline- and oxaliplatin-based chemotherapy, and *γ*-radiotherapy. The spatiotemporal sequence responsible or not for the stimulation of the immune system, dictating the success of cancer therapy, includes three phases: decision phase, processing phase, and effector phase [43]. 

In the decision phase, CRT early (within few hours) translocates on plasma membrane of dying tumor cells colocalizing in patches with PtdS in concomitance with ER stress response. CRT acts as an “eat me signal”, driving the engulfment of dying cancer cells and in parallel the uptake of tumor antigens by DCs [45]. The uptake, *via* a CRT-dependent manner, is ensured by loss/redistribution of CD47, a “don't eat me signal” that avoids the accidental phagocytosis of viable cells [157], occurring within 30 minutes after the induction of apoptosis [81] and hence displaying a pattern similar to CRT translocation. Late exposure on cell surface of HSP70 and 90 cooperates in the tumor antigen chaperoning and in the adhesion of tumor cells to DCs, respectively, [106, 158].

However, the DC-mediated uptake of tumor antigens is not sufficient to ignite immune response. In fact, the fusion antigen-containing phagosomes with lysosomes causes the destruction of antigens [159], that can be inhibited by recognition of DAMPs by TLRs localized on DCs surface [160]. Thus, other signals are required during processing phase to stimulate antigen processing. Among TLR4 ligands, the release of HMGB1, occurring within 18 hours after ICD induction, plays a key role. It has been suggested that the physical interplay between TLR4 and HMGB1 [49] triggers an immunogenic signal operating downstream of the DC-mediated antigen uptake that enables the optimal presentation of tumor antigens to T lymphocytes [73]. In addition, the antitumor immune response is controlled by NLR family, pyrin domain containing 3 (NLRP3) inflammasome [161]. ATP, released into the extracellular space during cellular stress, is the most abundant factor activating NLRP3 [162], that is essential for processing pro-IL-1*β* and secretion of IL-1*β* [163], that, in turn, primes CD4+ and CD8+ lymphocytes and elicits the antitumor response. ATP also functions as a “find me signal” promoting monocytes recruitment and activation [164].

In effector phase, matured and activated DCs elicit an IFN-*γ*-polarized T cell response, essential for efficient antitumor immunity [45, 49, 161].

**Figure 1:**
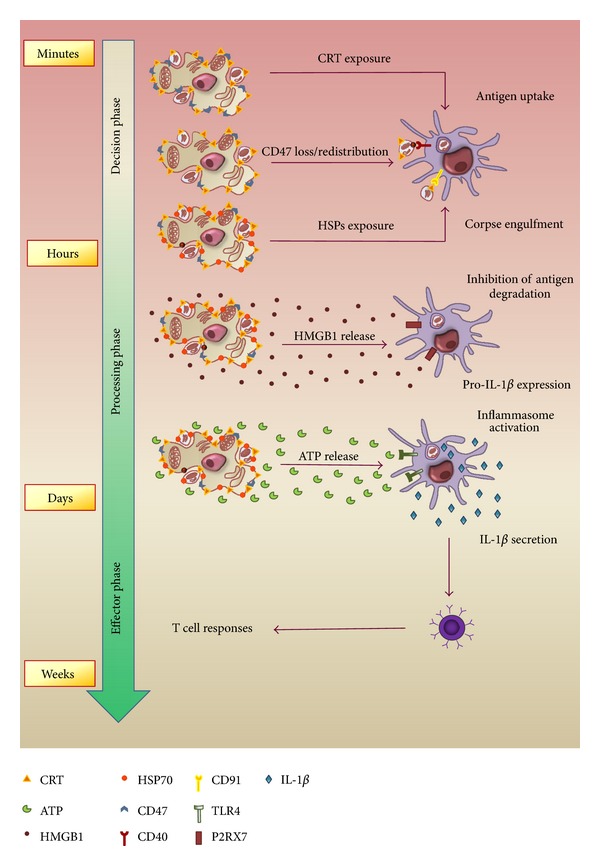
Spatiotemporal immunogenic signals delivery pattern. Decision phase, processing phase, and effector phase represent the spatiotemporal sequence determining the activation of immune system in *in vitro *conditions.

In Figure 1, we represent the temporal exposure and/or release of DAMPs and the relative response of immune cells in *in vitro* conditions.

## 5. The Response of Immune System to ****Photodynamic Treatment

The ability of PDT to induce activation of the immune system and specific antitumor immunity is a well-known phenomenon, recently reviewed in Pizova et al. [165]. Indeed, the ignition of anticancer immunity is as important as tumor cell death in designing an optimal cancer therapy [12, 40, 166]. In terms of ICD, various DAMPs can mediate antitumor immunity in PDT and a broad *spectrum* of photosensitizers localizing different subcellular organelles, especially ER, is very important in ICD triggering upon PDT [167].

### 5.1. How PDT Elicit Antitumor Immunity

Three mechanisms interplay to reduce and/or eradicate tumors after PDT: cancer cell death *via* ROS generation, ischemia of tumor area *via* destruction of tumor-associated vasculature depleting cancer cells of oxygen and nutrients, recruitment of inflammatory and immune mediators contributing to tumor destruction, and recognition of cancer cells *via* leukocytes invasion [168]. 

The triggered inflammatory responses are fundamental to achieve long-term tumor control [169]. 

The innate immunity against cancer is a step-by-step process involving initiation of inflammation, cytokine release, neutrophil infiltration of tumor site and neutrophilia and, finally, complement activation. On the other hand, acute inflammation can supply bioactive molecules to the tumor microenvironment, including growth factors that sustain proliferative signaling, survival factors that limit cell death, proangiogenic factors, extracellular matrix-modifying enzymes that facilitate angiogenesis, invasion, and metastasis, and inductive signals that support tumorigenesis [170–173]. Antitumor effect of cancer PDT involves both innate and adaptive immune system. 

PDT alters the tumor microenvironment by causing oxidative stress which triggers a vast array of signal pathways through TLRs, stimulating inflammation by expression of HSPs, NF*κ*B, and AP-1 [169]. The increased activity of these factors has been reported in several cancer cell lines photodynamically treated with different PSs. For example, NF*κ*B activation has been observed in L1210 mouse leukemia cells after Photofrin-PDT [174], in lymphocytes or monocytes infected with HIV-1 after proflavine-PDT [175], in human colon carcinoma cells phototreated with pyropheophorbide-a methyl ester (PPME) [176], and in human HL-60 cells after PDT with benzoporphyrin-derivative-(BPD-) verterporfin [177]. Similarly, AP-1 activation occurs in cervical carcinoma HeLa cells [178] and in epithelial PAM 212 cells [179] photosensitized with Photofrin.

NF*κ*B and AP-1 activation causes release by macrophages of different immunoregulatory and proinflammatory proteins, such as interleukins (IL-1*α*, -1*β*, -2, -6, -8, -11, -12, -15), tumor necrosis factor (TNF), chemokines (inflammatory protein IP-10, keratinocytes-derived chemokines KC, Macrophage Inflammatory Proteins MIP-1*α* and *β*, MIP-2, eotaxin, Methyl-accepting Chemotaxis Protein MCP-1, Regulated on Activation Normal T cell Expressed and Secreted, RANTES), and interferons (IFN-*α* and *β*) (reviewed in [169]). 

Other cell types eliciting innate immunity in PDT are neutrophils. They play a fundamental role both in the direct killing of tumor cells and in the activation of other immune cells. Moreover, they are also a source of proinflammatory mediators [180]. Neutrophils do not only accumulate in PDT-treated tumors, but they are also present in the blood of the host (so-called neutrophilia) [181]. Cecic et al. [182] reported that, in mammary carcinoma EMT6 tumors, Photofrin-PDT induces in the host mice a significant increment of neutrophils in blood persisting for at least 10 hours after treatment. Also Gollnick et al., [34] report that 2-[1-hexyloxyethyl]-2-devinyl pyropheophorbide-*α* (HPPH)-mediated-PDT causes neutrophil migration into the tumor area as well as Krosl and colleagues [183] report a 200-fold rise in neutrophils in the cellular infiltrate in SCCVII tumor treated with Photofrin-PDT. Finally, treatment of rat rabdomyosarcoma tumors with 5-aminolevulinic acid (5-ALA)-PDT induces a blood neutrophils increase during the first few days after illumination [184].

Thus, PDT prompts a powerful acute inflammation leading to activation of complement cascade likely by the alternative pathway [180, 182, 185, 186]. In particular, *in vitro* studies indicate that PDT induces fixation of complement C3 protein to tumor cells [187] that, in turn, marks the cells to be destructed by the innate immune system [188–190]. The complement system can directly promote T-cell mediate response, playing a role in the adaptive immunity [191] carried out by antigen-specific B and T cells.

The growth inhibition of murine EMT6 tumors dependent on the presence of CD8+ T cells has been demonstrated after Photofrin-PDT by Kabingu and coworkers [192]. Preise et al. [38], by transferring CD8+ and CD4+ T cells from mice survived to cancer 3 months after vascular targeted PDT with bacteriochlorophyll derivative WST11, found that the transferred mice are protected from subsequent challenge with viable cancer cells. Similar results have been obtained in human studies. For example, Thong et al. [193] demonstrated increased CD8+ T cell infiltration into multifocal angiosarcoma of the head and neck carcinoma area phototreated with Fotolon, a PS comprising 1 : 1 chlorin e6 and polyvinylpyrrolidone. Photodynamic treatment with Photofrin or 5-ALA in patients with basal cell carcinoma provides an enhanced recognition of MHC-I-antigen complexes by immune cells and the activation of tumor specific CD8+ T cells [194].

The activity of CD8+ cells upon PDT is correlated to the presence of tumor antigens eliciting a potent antitumor effect. In fact, Mroz and coworkers [195], demonstrated that BPD-PDT can induce a strong antigen specific immune response capable to incite the memory immunity enabling BALB/c mice to reject a tumor rechallenge obtained with the same antigen positive tumor from which they were cured. The importance of tumor antigen presence is confirmed by the observation that BPD-PDT low level distant metastasis destruction correlates with a loss of tumor antigen expression. 

It is worth mentioning that, certain PDT regimens systematically suppress immune reactivity [169]. Cutaneous Photofrin-PDT induces elevated levels of systemic IL-10, correlating to a prolonged suppression of contact ipersensitivity (CHS) reaction of at least 28 days following treatment. Since the major effector cell in CHS is the IFN-*γ* secreting CD8+ cell and IL-10 suppresses cell-mediated immune response *via* inhibition of CD4+ cells activation, it is possible that PDT negatively influences the CD4+ or CD8+ development. Moreover, the inhibition of CD4+ or CD8+ activity can be caused by induction of systemic IL-4 [196]. Yusuf et al. [197] demonstrated that silicon phtalocyanine (Pc4)-PDT causes immunosuppression in cancer ill mice by involving of CD4+ and CD8+ T cells. Moreover, immunosuppression can be adoptively transferred with spleen cells from Pc4-PDT treated donor mice to syngenic naive recipients and it is primarily mediated by T cells, although macrophages were also found to partecipate. Among CD4+ cells, a special population that functionally suppresses an immune response is Tregs. They mediate their immunosuppressive effects by multiple pathways [198]. Particularly, Tregs express TGF-*β*, an immunosuppressive cytokine [199] participating in further proliferation of Tregs [200], and suppress DCs activation [201]. Castano and collegues [202] observed that Tregs can be depleted by cyclophosphamide (CY) that, in combination with PBD-PDT, leads a long-term J774 reticulum cell sarcoma cure and resistance to tumor rechallenge. 

### 5.2. Involvement of ICD in Photodynamic Cancer Therapy

The link between PDT and HSPs, and especially HSP70, the best characterized DAMPs in PDT, [203] is already known as well as the immunogenicity of PDT treatment and its ability to elicit an antitumor immunity [204]. 

PDT is able to trigger the main types of cell death, that is, apoptosis, autophagic cell death, and necrosis [26]. It is interesting to understand if there exists a relation between PDT-induced cell death and DAMPs and between DAMPs and ICD. In case of necrosis, DAMPs *spectrum* does not change relatively to agents inducing them, including PDT. For example, the release of HMGB1 has been demonstrated both in serum of Photofrin-treated mice with subcutaneous Lewis Lung Carcinoma (LLC) and in LLC Photofrin-treated cells [205]. On the other hand, HMGB1 has been reported to be the only DAMP involved in autophagy [144–146], but nothing is known about the DAMPs associated with autophagic cell death induced by photodynamic treatment. Moreover, PDT-induced apoptotic cells have been predominantly associated with HSPs [204] and only recently it has been suggested the involvement of CRT and ATP in apoptotic cell death upon photodynamic sensitization [70].

Interestingly, we found, for the first time, that Rose Bengale Acetate (RBAc) is able to induce the release and translocation of HSP70 and 90 and the exposure of CRT on plasma membrane of both apoptotic and autophagic RBAc-PDT induced HeLa cells (unpublished data). However, it is still under investigation the involvement of these DAMPs in RBAc-PDT induced immunogenicity.

**Figure 2:**
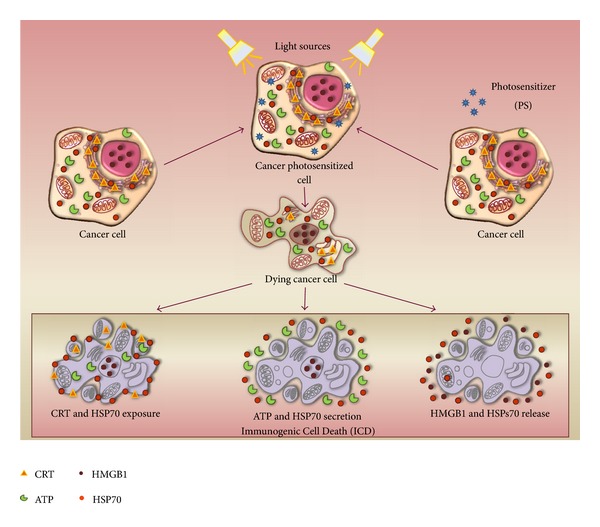
Molecular mechanisms of PDT-induced Immunogenic Cell Death (ICD). The three main processes characterizing the ICD are the preapoptotic cell surface exposure of calreticulin (CRT) and HSP70, the secretion of ATP and HSP70, and the postapoptotic release of HMGB1 and HSP70.

The DAMPs *spectrum* observed in PDT is reported in Figure 2.

In the following section, the link between DAMPs-associated PDT and their involvement in the elicitation of immune cells have been discussed in detail.

**Interplay between DAMPs Involved in PDT-Induced Cell Death and Immunogenicity**

The best characterized DAMPs involved in PDT-triggered cell death able to confer immunogenicity are HSPs proteins and especially HSP70, as described for squamous cells carcinoma SCCVII [203], murine mammary tumor cells C127 [206] during Photofrin-PDT, EMT6 cells during meso-tetrahydroxyphenyl chlorin (mTHPS, Foscan)-PDT [207] and glioblastoma cell lines U87 and U251 during 5-ALA-PDT [208].

It has been observed that almost instantaneously HSP70 can be translocated onto the outer leaflet of plasma membrane of SCCVII cells apoptotically committed with Photofrin-based PDT. The authors reported that several other HSPs, that is, HSP60 and Glucose regulated Protein 94 (GRP94) are also exposed at the surface of tumor SCCVII cells. A fraction of HSP70 is promptly (within 1 hour) released by cells after high treatment doses, whereas lower PDT doses induce a HSP70 release at later time intervals, suggesting that the release is a consequence of membrane permeabilization upon necrosis. The induction of cell surface expression and release of HSPs stimulate macrophages coincubated with PDT-treated SCCVII cells to produce TNF-*α*. This study also suggests that DAMPs exposed onto the surface in response to PDT stress based on *in vitro* or *in vivo* settings, are probably related to tumor microenvironment. In fact, when authors induce SCCVII tumor in mice, they observe that cancer cells expose GRP78 rather than HSP60 and GRP94 [203]. Moreover, ecto-HSP70 participates in the opsonization of cancer cells by C3 complement protein [209]. Similarly, Zhou and coworkers [206] demonstrated that HSP70 secreted and released by C127 cells induced to apoptosis by Photofrin-PDT orchestrates an immunological regulatory mechanism towards murine Raw 264.7 macrophages. In fact, macrophages incubated with apoptotic cells as well as necrotic tumor cells showed a high level of TNF-*α* secretion. Also EMT6 cells photosensitized with Foscan expose and release HSP70 resulting in long-term tumor growth control [207]. In 5-ALA spheroids of glioblastoma cell lines U87 and U251, HSP70 is expressed on the surface of cancerous cells and induces both attraction and maturation of DCs and antigen uptake by upregulation of CD83 and costimulatory molecules as well as increasing T-cell stimulatory activity of DCs [208].

Recently, experiments performed in the Agostinis laboratory reveal that the CRT exposure and ATP secretion during PDT elicit ICD, adding Hypericin-(Hyp-) based PDT to the list of ICD inducers [70, 210].

Hyp-PDT causes, soon after 30 minutes after irradiation, the precocious exposure on surface of T24 human bladder carcinoma and colon carcinoma CT26 cells of both CRT and HSP70 [210]; moreover, T24 cells secrete ATP in response to Hyp-PDT [70]. Ecto-CRT exposure depends on the PDT dose, both in PS concentration and in light fluence; surprisingly, conversely to the literature data reporting cotranslocation of ERp57 and CRT on plasma membrane, after Hyp-PDT, T24 cells expose CRT in the absence of ERp57 and it does not require eIF2*α* phosphorylation, caspase 8 activity, and increased cytosolic Ca^2+^ concentration [70, 210]. In terms of immune cells ignition, only ecto-CRT influences phagocytosis of T24 and Ct26 dead cells. In fact, T24 cells succumbing to Hyp-PDT were engulfed by murine Mf4/4 macrophages and human DCs [70]; similarly, CT26 Hyp-PDT photokilled cells were preferentially phagocytosed by JAWSII murine dendritic cells [210]. Moreover, CT26 Hyp-PDT photokilled cells immunized syngenic BALB/c mice against a recidive in the presence of living CT26 cancer cells [70]. DCs cultured in the presence of Hyp-PDT treated T24 cells produce high levels of Nitric Oxide (NO) and IL-1*β*, but they do not secrete the anti-inflammatory IL-10 [70]. Since IL-1*β* is involved in polarization of IFN-*γ*- producing antineoplastic CD8+ T cells [211], Garg et al. [70] found the antitumor immune response elicited by Hyp-PDT. The authors [70] also suggest the processes and molecules sustaining CRT exposure, that is, ROS production, class I phosphoinositide-3-kinase (PI3K) activation, actin cytoskeleton, ER-to-Golgi anterograde transport, PERK, Bax and Bak proapoptotic proteins and CRT cell surface receptor CD91, and ATP release, that is, ER-to-Golgi anterograde transport, PI3K and PERK.

## 6. Concluding Remarks

The knowledge of DAMPs involved in cell death inducing cancer settings could help in the prompt choice of the better therapeutic design for a particular cancer condition. In fact, cancer therapies associated with DAMPs expression, in term of both exposure on plasma membrane and release on extracellular environment, have been shown to be able to fuse efficient cell death induction and activation of antitumor immune response. DAMPs can be also exploited as tool to mark disease's stage and to identify the extent of inflammation associated with the disease. Further, they can be used to prepare highly immunogenic vaccines. Due to its uniqueness to efficiently induce cell demise, antitumor effect by involving both innate and adaptive immune system and DAMPs expression, PDT is very promising to optimize cancer protocol. In fact, by inducing ICD, PDT could be capable to counteract cancer recurrence by instructing immune system.

In this context, it is a priority for the future oncology practice to clarify the molecular mechanisms associated with the immune response triggered by immunogenic tumor cell death.
